# Complete excision and soft tissue augmentation after recurrence of a peripheral ossifying fibroma as a pyogenic granuloma: A case report

**DOI:** 10.34172/japid.2020.016

**Published:** 2020-12-05

**Authors:** Ramtin Chitsazha, Masoumeh Faramarzi, Negin Firouzi

**Affiliations:** ^1^Department of Periodontics, Faculty of Dentistry, Tabriz University of Medical Sciences, Tabriz, Iran; ^2^Department of Endodontics, Faculty of Dentistry, Tabriz University of Medical Sciences, Tabriz, Iran

**Keywords:** Case report, Excisional biopsy, Free gingival graft, Reactive lesion

## Abstract

Reactive lesions of soft tissue are common oral lesions that are usually non-neoplastic growths without pain; however, they can interfere with oral hygiene and plaque control, and if present in the anterior segment, they can cause esthetics problems. In this case, there was a reactive lesion at the gingiva of the left maxillary central incisor area. The particular consideration about this case was the recurrence of a peripheral ossifying fibroma as a pyogenic granuloma, indicating the association between reactive gingival lesions. The history of several recurrences due to incomplete removal shows the importance of complete excision for preventing recurrence. Therefore, the lesion was completely excised, and the mucogingival defect was successfully augmented with periodontal plastic surgery.

## Introduction

Dentists during routine examinations might face peripheral reactive lesions of soft tissues. Since oral mucosa is under the continual influence of different irritants, it exhibits a range of disorders, injuries, inflammation, and neoplastic conditions.^1,2^

Reactive lesions are common on the gingiva and tend to be not painful, without neoplastic growths, but often interfere with plaque control and cause patient discomfort. Most of these lesions are discovered between the fifth and sixth decades of life with a predilection for females.^3^

Since these lesions might be present for a long time, they might be traumatized and ulcerated. After treatment, the recurrence rate is 5–20% based on the diagnosis, complete removal, and the elimination of irritating factors related to their etiology.^4^ Histologic analysis is necessary for diagnosing these lesions because they are often clinically indistinguishable, but due to diagnosis, the recurrence rate might be different.The treatment of reactive lesions is surgical excision, which can be carried out by a scalpel blade, laser, or electrosurgery. A complete treatment should also consider determining the main etiology through a precise clinical evaluation and elimination of traumatic habits or any other etiological factors that led to the reactive lesion. Dental hygiene maintenance with professional follow-up care should reduce the recurrence prevalence for most types of gingival lesions.^5^

The classification of common gingival overgrowth lesions is as follows: pyogenic granuloma (PG), peripheral ossifying fibroma (POF), peripheral fibroma (PF), and peripheral giant cell granuloma (PGCG).^6^

These lesions are not regarded as neoplasms, and histopathological analysis can identify their diagnosis. They are considered reactive lesions and occur due to local irritants such as trauma, faulty restorations, dental plaque, calculus, and iatrogenic factors.

Since these lesions’ clinical findings are similar, a histological analysis must confirm the definite diagnosis of the lesion.

However, they are benign in nature; incomplete removal of the lesion or persistence of the local irritants leads to their tendency to recur. Hence, the treatment is surgical excision and complete removal to reduce the recurrence rate.^7^

Furthermore, the consequence of complete excision of the lesion might be a periodontal defect without keratinized tissue, with root exposure and esthetic problems.^8,9^

Thus, the ability of the surgeon to get a decent result involves augmentation and repair of the mucogingival defect and complete excision of the lesion. All of these can be performed during one surgical procedure with proper planning.

This paper presents the management and treatment of a reactive lesion of soft tissue in the gingiva of the left maxillary central incisor with a history of several recurrence episodes due to incomplete removal, compelling us to perform a complete excision and consequently a free gingival graft to regenerate the excised tissue.

## Case report

**Figure 1:**
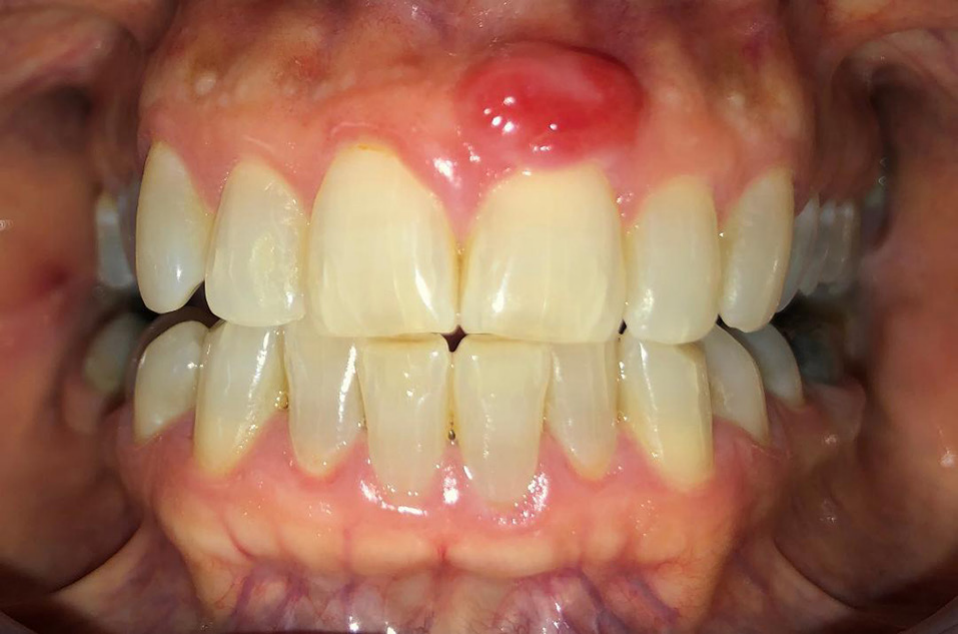
Soft tissue lesion, preoperative photograph

A 52-year-old female patient referred to the Department of Periodontics at Tabriz University of Medical Sciences, complaining of esthetic problems due to a soft tissue lesion located on the gingiva of left maxillary central incisor, without pain, with a history of almost seven years and several attempts of incomplete removal and recurrence (Figure 1).

The last biopsy had led to a diagnosis of a peripheral ossifying fibroma. Recurrence had occurred due to incomplete removal of the lesion. Intraoral examination revealed a lesion measuring approximately 1×0.6×0.5 cm, which was nodular, exophytic, and sessile. The radiographic image showed normal structures and no related changes.

**Figure 2:**
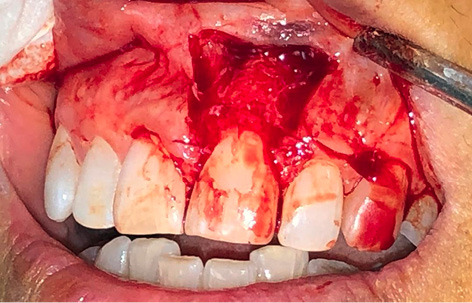
Periodontal defect after excision and curettage (recipient site).

The surgery was performed under local anesthesia with 2% lidocaine and epinephrine at a concentration of 1:100,000. The excision of the lesion was instituted with a #15C blade. The lesion and the involved periodontal tissue were removed, followed by thorough curettage of the underlying bone and root planing of the tooth (Figure 2).

After the excision, the lesion was taken with fine tissue forceps and transferred to a specimen bottle containing 10% neutral buffered formalin with almost 20 times the sample’s volume. A biopsy data sheet was completed, and the specimen was delivered to the laboratory.

A free gingival graft was harvested from the palate to place in the exposed area to regenerate the lost periodontal tissue due to the excision.

**Figure 3:**
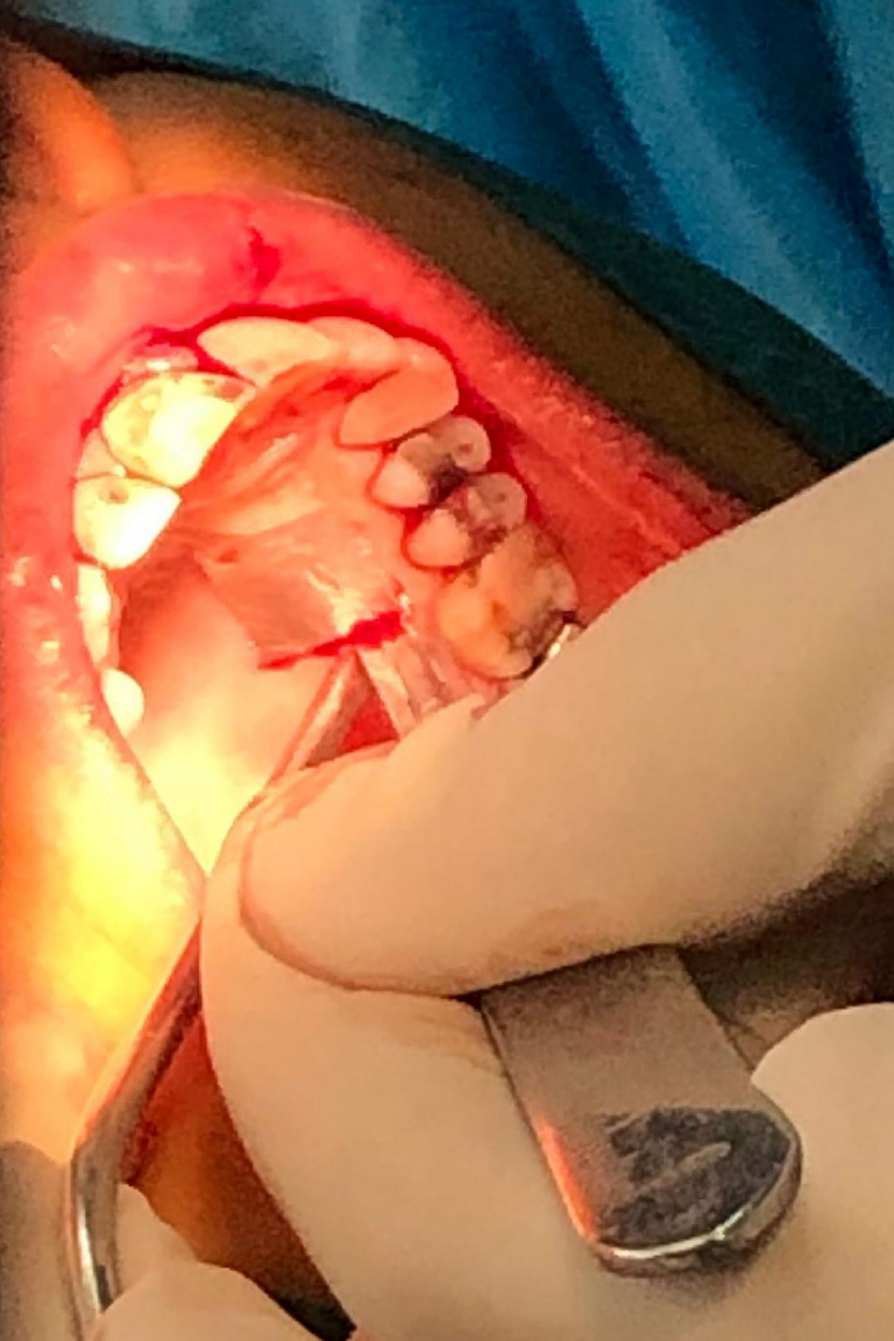
Harvesting free gingival graft from donor site.

A sterile aluminum foil template of the recipient site was made to be used as a pattern for harvesting the graft from the palate. The template was placed over the donor site, and a shallow incision was made around it perpendicular to the tissue with a #15 blade (#15 blade was selected due to its wider tip because we only needed the tip of the blade) (Figure 3).

The second incision was made in several steps to ensure inserting the blade perpendicular to the first incision to achieve an equal thickness all over the graft.

The graft consisted of epithelium and a thin layer of underlying connective tissue with a thickness of 1.5 mm.

**Figure 4:**
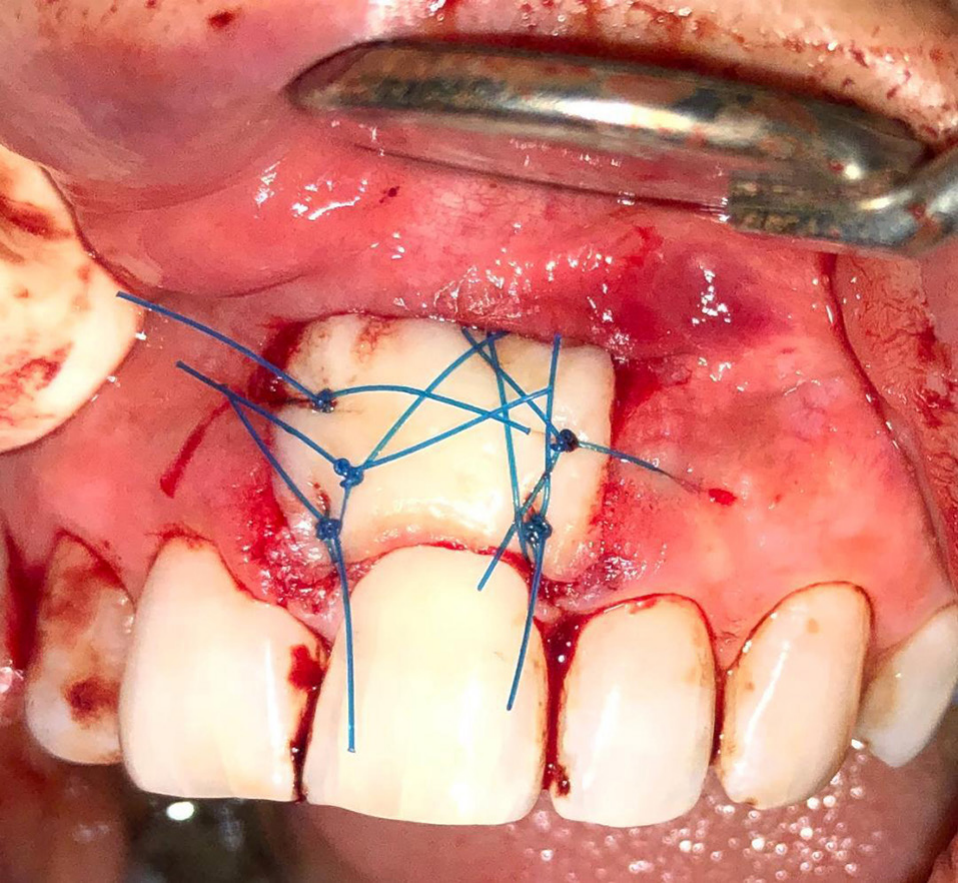
Adaptation and suturing of the graft in the recipient zone.

After the graft was separated, it was immediately transferred, the excess clot was removed from the recipient site, and the graft was positioned and firmly adapted with non-absorbable monofilament polypropylene 4-0 sutures (Figure 4).

**Figure 5:**
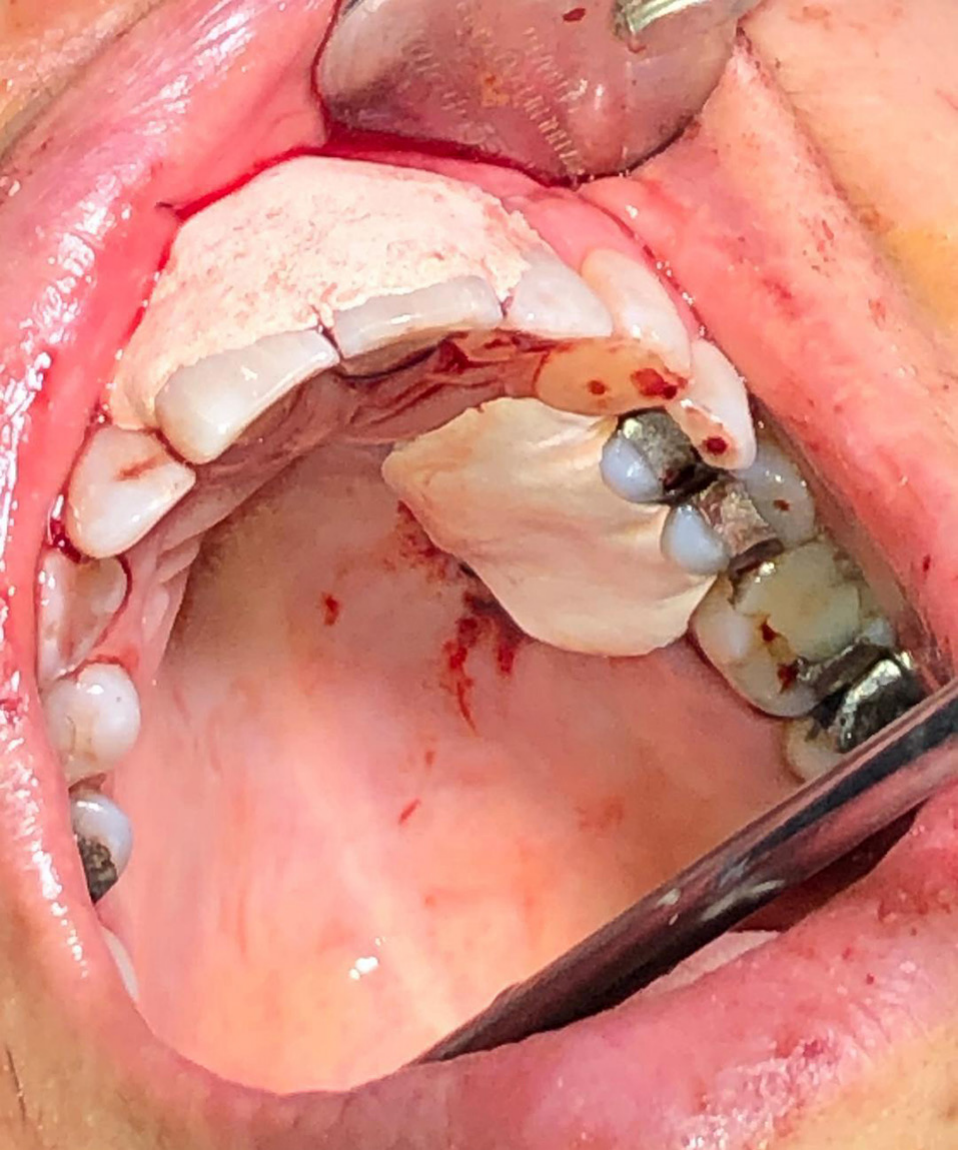
Periodontal dressing.

The recipient and the donor site were covered with a eugenol-free periodontal dressing (Coe-pak, GC, USA) to comfort the patient and reduce postoperative complications (Figure 5).

After providing postsurgical instructions, the following medications were prescribed:

Amoxicillin 500 mg q8hr for one week, Ibuprofen 400 mg q6hr PRN, and 0.12% chlorhexidine mouthwash q12hr for two weeks.

Ten days later, the sutures and periodontal dressing were removed at the first follow-up, and recall arrangement continued at 2 weeks and 1, 2, and 6 months after the first follow-up.

The pathological analysis confirmed the reactive nature of the lesion and reported a highly vascular proliferation in connective tissue resembling granulation tissue, covered by hyperplastic stratified squamous epithelium, diagnosed as a “pyogenic granuloma.”

## Discussion

Reactive lesions of soft tissue are common oral lesions that are clinically indistinguishable from one another; therefore, it is important to differentiate them using histological analysis.

Gingival tissues are exposed to constant irritation of bacterial plaque and biofilm. Reactive lesions generally arise from the periodontal ligament and connective tissue; therefore, the gingiva is the most common reactive lesions site.^1^ Also, gingival tissues in the anterior mandible and maxilla are the most affected areas that might cause esthetic problems.^10^ Complete removal of the lesion and the elimination of irritating factors and their etiology are necessary for reducing the recurrence rate.^4^

There are few articles indicating an interrelationship between these lesions. For example, POF and PGCG are site-specific lesions arising from the periodontal ligament and share histological features, such as abnormal connective tissue proliferation with varying amounts of giant cells and focal osteogenesis. However, PGCG is composed of relatively more immature and loose components compared to POF.^11^

Ogbureke et al^12^ reported a case of PGCG with extensive osseous metaplasia or a hybrid PGCG-POF.This case raises the question of whether non-neoplastic proliferative lesions are part of a disease spectrum or whether some of these lesions are true hybrid lesions.

Furthermore, there are few case reports indicating an interrelationship between PG and POF, similar to our case.

Prasad et al^13^ recommended that PG and POF might be different stages of the same pathology. Sridhar et al^14^ also reported a case of PG in the mandibular anterior segment, which was surgically excised but recurred after a year as a POF in the same segment. The authors concluded that regular follow-up is very important to avoid recurrence. Singhal et al^15^ reported the recurrence of a POF as a PG within one week. This case emphasizes complete excision to prevent a recurrence.

In our case, unacceptable esthetic was the patient’s chief complaint; therefore, we prepared a dual-purpose treatment plan, which included excision of the lesion, along with the regeneration of the anterior defect caused by excisional biopsy. In this treatment, excisional biopsy and free gingival graft were performed during one surgical procedure.

Since there was a history of several recurrences due to incomplete removal of the lesion in the past, complete removal and thorough curettage of the underlying bone were necessary for treatment success.

**Figure 6:**
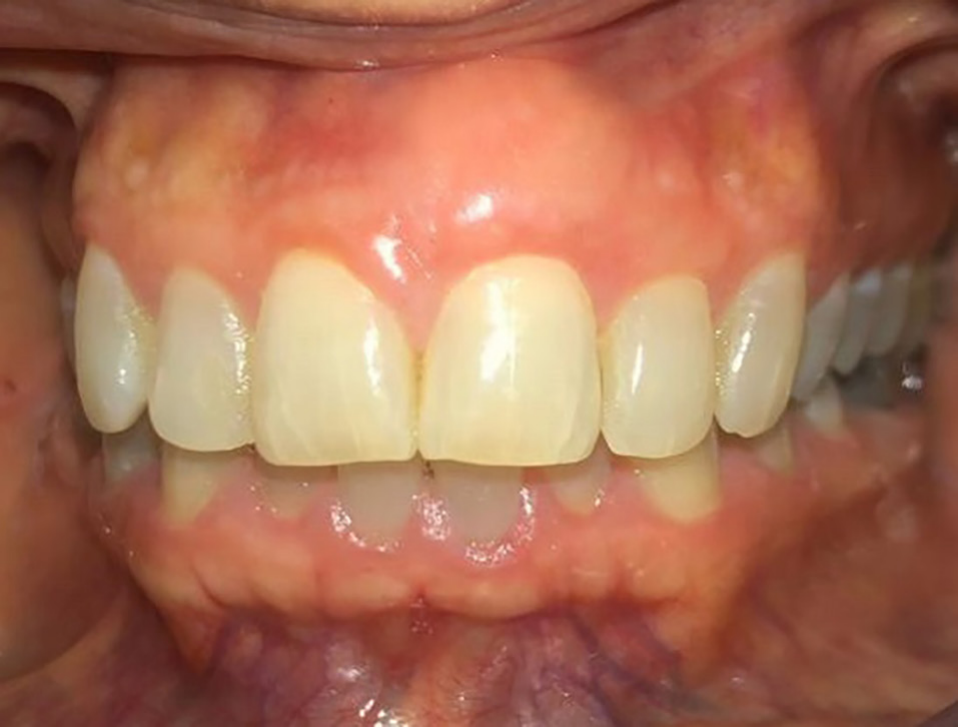
Follow-up after 6 month.

In our case, after placing the graft, the defect resulting from the excisional biopsy was fully reconstructed. The patient was satisfied with the esthetic appearance and without any inflammation or bleeding on probing after six months of follow-up (Figure 6).

## Conclusion

Reactive lesions are common oral lesions, and the treatment for all the common reactive lesions includes surgical excision and complete removal to prevent a recurrence, but it might result in a mucogingival defect.

Therefore, this case report highlights the augmentation and repair of the mucogingival defect and the lesion’s complete excision to achieve satisfactory results. All of these can be performed during one surgical procedure with proper planning.

## Authors’ Contributions

RC carried out the procedures; MF carried out the procedures and supervised the project; NF prepared the manuscript, searched the literature. and designed the figures. All authors have read and approved the final manuscript.

## Competing interests

The authors state no competing interests.

## Ethics Approval

The patient signed a consent form before surgery and publishing this report.

## References

[1] Kadeh H, Saravani S, Tajik M (2015). Reactive hyperplastic lesions of the oral cavity. Iran J Otorhinolaryngol.

[2] Palmeira ARBLS, Florêncio AG, Silva Filho JP, da Silva UH, Araújo NS (2013). Non neoplastic proliferative lesions: a ten-year retrospective study. Rev Gaucha Odontol.

[3] Cruz GM da, Trezena S, Pêgo SPB, Paranaíba LMR, Martelli DRB, Melo Filho MR de (2019). Profile evaluation of patients diagnosed with non-neoplastic proliferative lesions in a dentistry clinic. Brazilian J Oral Sci.

[4] Rossmann JA (2011). Reactive Lesions of the Gingiva: Diagnosis and Treatment Options. Open Pathol J.

[5] Babu B, Hallikeri K (2017). Reactive lesions of oral cavity: A retrospective study of 659 cases. J Indian Soc Periodontol.

[6] Neville BW, Damm DD, Allen CM, Bouquot JE (2009). Oral and Maxillofacial Pathology.

[7] Effiom OA, Adeyemo WL, Soyele OO (2011). Focal reactive lesions of the gingiva: an analysis of 314 cases at a tertiary health institution in Nigeria. Niger Med J.

[8] Henriques PSG, Okajima LS, Nunes MP, Montalli VAM (2016). Coverage Root after Removing Peripheral Ossifying Fibroma: 5-Year Follow-Up Case Report. Case Rep Dent.

[9] Chaudhari P A, Nasir S, Gulati R, Ratre M S (2019). The role of periodontal plastic surgery in the aesthetic management of localized gingival overgrowth. IP Int J Periodontol Implantol.

[10] Hunasgi S, Koneru A, Vanishree M, Manvikar V (2017). Assessment of reactive gingival lesions of oral cavity: A histopathological study. J Oral Maxillofac Pathol.

[11] Moeiny P, Shabani S, Vatankhah M, bakhshi A, Zameni M (2019). Report of a Case of Peripheral Giant Cell Granuloma in Anterior Maxillary Region. J Res Dent Maxillofac Sci.

[12] Ogbureke EI, Vigneswaran N, Seals M, Frey G, Johnson CD, Ogbureke KUE (2015). A peripheral giant cell granuloma with extensive osseous metaplasia or a hybrid peripheral giant cell granuloma-peripheral ossifying fibroma: A case report. J Med Case Rep.

[13] Prasad S, Reddy SB, Patil SR, Kalburgi NB, Puranik RS (2008 Mar). Peripheral ossifying fibroma and pyogenic granuloma Are they interrelated?. N Y State Dent J.

[14] Sridhar R, Wanjari S, Kanteshwari IK (2012). Interrelationship between pyogenic granuloma and peripheral ossifying fibroma: a case report. J Dent Hyg.

[15] Singhal A, Arora R, Sharma A (2016). Recurrence of a Peripheral Ossifying Fibroma as a Pyogenic Granuloma Within 1 Week: A Case Report. Clin Adv Periodontics.

